# Artificial Intelligence and Agency: Tie-breaking in AI Decision-Making

**DOI:** 10.1007/s11948-024-00476-2

**Published:** 2024-03-29

**Authors:** Danielle Swanepoel, Daniel Corks

**Affiliations:** SolBridge International School of Business, 128 Uam-ro, Samseong-dong, Dong-gu, Daejeon, Korea

**Keywords:** Artificial intelligence, Agency, Decision-making, Rationality, Tie-breaking

## Abstract

Determining the agency-status of machines and AI has never been more pressing. As we progress into a future where humans and machines more closely co-exist, understanding hallmark features of agency affords us the ability to develop policy and narratives which cater to both humans and machines. This paper maintains that decision-making processes largely underpin agential action, and that in most instances, these processes yield good results in terms of making good choices. However, in some instances, when faced with two (or more) choices, an agent may find themselves with equal reasons to choose either - thus being presented with a tie. This paper argues that in the event of a tie, the ability to create a voluntarist reason is a hallmark feature of agency, and second, that AI, through current tie-breaking mechanisms does not have this ability, and thus fails at this particular feature of agency.

## Introduction

Agency is often described as the ability for an individual or entity to perform intentional, autonomous actions. Underpinning these actions are decision-making processes. The difference between a mere act and an action is that a choice is made in one and not the other. The act of jerking my leg in my sleep is not a choice, but the action of kicking another individual so that they can move out of my way is a choice. Decision-making is foundational to rational agency, often serving as the bedrock for intentional, autonomous action. Through decision-making, an agent is able to determine the best possible means to achieve desired goals. Daily, human agents are faced with hundreds of decisions ranging from what to wear to work to which career to pursue. Serving these decision-making processes are desires, drives, motivations, norms, rules, and principles (among others). These decision-making processes appear intuitive, heuristic, and evolutionary in nature; often seeming as if human agents become increasingly better at decisions as they learn from experiences.

Agents, when faced with multiple choices, may find some decisions more difficult than others, especially if the decision is an important one and the choices are multiple. In these instances, agents will actively sift through reasons to opt for one choice over another. Almost always, there are more reasons to support one decision over another which allows for the choice to be made. In more complex situations, an agent may take longer to sort through the reasons, but almost always, reasons for one will outweigh the other. Arguably, an agent who opts for a choice that yields less reasons to choose it than the other, would not be making a correct, or good, decision.

In rare situations however, an agent may encounter two choices where there are equal reasons to do both, or that both are equally undesirable. Again, this can happen over unimportant decisions, such as whether to add dairy milk or oat milk to your coffee, or for very important decisions, such as which life insurance to invest in. In instances where there are equal reasons to choose either, an agent must, or should, find a way out of the predicament or be accused of not being actualizing agency. Some theorists view this type of situation as a challenge to rational choice theory (Champagne, 2015; Stone, 2014) while others (Chang, 2009) view it as an opportunity to fully embrace agency. Explored in section “Voluntarist Reasons” of this paper, Chang (2009) argues that in this instance, an individual can arbitrarily choose (through flipping a coin perhaps), or can re-examine the reasons *ad infinitum*, or can *will* a reason (voluntarist reason) to be the reason that supports one choice over another. Arguably, the ability to *will* a reason, and thus *break a tie* in the decision-making process, is another hallmark feature of agency.

Recognizing that decision-making is foundational to action, and that autonomous, intentional action is a hallmark feature of agency, this paper explores the agency-status of AI. Of special interest is the capacity of AI to break ties; this capacity, at present, appears unique to human agency and its absence in AI should prompt AI researchers and ethicists to explore this further. To explore the relationship between tie-breaking and agency and agency in AI, this paper is divided into six sections. section “Agency in AI, and in Humans” unpacks the status of agency in AI and the status of agency in human beings. In section “Voluntarist Reasons”, we examine Ruth Chang’s contribution to the story of agency, by looking at how agents are able to will a reason to be that which affords them the opportunity to choose one option over another in the event of a tie. In section “AI and Tie-Breaking”, we explore the decision-making process of AI in the event of a tie. In section “The Impacts of AI Tie-breaking on the Agency-status of Machines”, we show how AI is not, currently, in the position to will reasons and thus, may not satisfy this particular feature of agency.

## Agency in AI, and in Humans

### The State of Agency in AI

Considerable research has been done over the last few decades concerning agency and machines (AI). Determining agency-status is an important task for AI ethicists as agency-status informs legislation, policy, moral obligation, responsibility, accountability, and punishment (among other things). According to Rabiza (2022), inquiry into AI agency can take two forms: point notions of agency and network approaches to agency (referring a network of actors– both Human and AI– and the relationships they share as indicative of agency-status). While the latter is certainly interesting, for the purpose of this paper, we shall focus primarily on the former. Point notions of agency seek out specific criteria or traits that are constitutive of agency or are hallmark features of agency. Swanepoel (2021a) identifies four features of agency in what she calls “Common Ground Agency”. Common Ground Agency holds that (i) deliberative self-reflection, (ii) awareness of self in time, (iii) critical awareness of environment, and (iv) norm violation are minimal criteria for agency. She offers an analysis of these criteria under an AI-friendly banner (thus actively addressing the worries that agency is often too anthropocentric in its definition) and concludes that AI falls short in at least two of these criteria.

Some theorists have examined the agency status of AI by examining the potential for normativity in AI and have either identified normativity as a feature of agency or have explored ways in which AI can be infused with normativity in their decision-making (Anderson et al., 2006; Barandiaran et al., 2009; Bringsjord et al., 2006; Langley, 2019). Notably, the fourth criteria in Common Ground Agency explores the possibility of normativity in AI and this is one of the criteria where it does not hold (Swanepoel, 2021a, b).

Others have identified intentionality as a hallmark feature of agency for AI. Papagni and Koeszegi (2021) “defend the thesis that approaching these artificial agents ‘as if’ they have intentions and forms of social, goal-oriented rationality the only way to deal with their complexity on a daily basis”. Problematically though, treating AI “as if” it has these features is not proof that these features exist in these entities– but perhaps is an interesting way to operationalize these discussions (since blackbox notions only go so far). The strongest proponent of intentionality is Dennett (1981, 1988, 1989). The intentional stance (Dennett), simply put, is a predictive tool that can be used to predict the behaviour of rational agents based on their intentions, beliefs, and desires. Of course, to argue that intentionality is a hallmark feature of rationality– and to argue that AI could be a rational agent– is to argue for the existence of intentions, beliefs, and desires in AI, and like Rovane (2004, p. 321) argues, the intentional stance is not enough; a rational being “must see that it ought to be rational”. Johnson (2006) does not believe this is a requirement; she argues that AI does indeed have intentionality: “the intentionality put into them by intentional acts of their designers” (Johnson, 2006, p. 201). This, however, challenges the notion of autonomy and norm-adherence or even norm-recognition (Kant, 2004; Moore, 2011; Swanepoel, 2021b), which most would argue are foundational to agency.

While all features above are noteworthy and are considered hallmark features of agency, this paper argues that a particular element (that of tie-breaking through voluntarist reasons) of decision-making should also be added to the list of hallmark features of agency. We pose a challenge for AI researchers to include this element of decision making in their repertoire of criteria needed to determine the agency status of AI.

### The State of Agency in Humans

A caveat before moving forward: the authors do not claim that human decision-making is always rational, nor do we argue that human beings are archetypical agents. We do however believe that humans (more generally) are able to better exhibit agency than most other entities. We also understand the restrictions that anthropocentric definitions pose in discussions such as these– it is certainly the case that definitions of agency and the features that are espoused therein have been almost always aimed at discussions about human agency^1^. We also think that setting a hallmark feature of agency in something like tie-breaking through voluntarist reasons is a fair criterion that can be applied equally to humans and to machines.

Human agents are those that perform autonomous, intentional actions.^2^ Alongside the ability to perform autonomous, intentional actions, human agents are also held accountable for their actions and are responsible for the decisions they make. What is often perceived as the hallmark of rational agency is the capacity or ability to successfully engage in means-ends reasoning (Korsgaard, 2009; Railton, 2003; Velleman, 2009).

According to Velleman (2000), the standard story of agency is not complete. Velleman argues that under the general accounts of agency (such as the neo-Humean, neo-Aristotelian accounts), the accounts make it seem that actions happen to agents rather than agents being authors of those actions. For Velleman, an agent’s actions are aimed at *knowing* what she is doing and doing what makes sense. Velleman provides the example of Frankenstein being tasked to infuse his monster with agency, it would mean designing the monster in a way that it would “gravitate towards knowing what they’re doing, and they will only do those things which they have made up their minds that they’re going to do, and so they will act by choice” (Velleman, 2000, p. 26). Agents then, are those for which actions that are most intelligible are those that one would typically feel driven to choose.

As we are driven to choose, human agents (and AI, to an extent) largely depend on what is valuable (or considered valuable) to assist in determining goals and identifying means to achieve those goals. Value can be conceptualized in terms of goodness or desirability (Foot, 1958; Nagel, 1989; Schroeder, 2021), such that an action, object, relationship, or event, can be considered valuable if it is a good thing to do, have, or be in. If actions hold little value, a person is less likely to undertake that action. Value can be based on several things: utility, goodness, and preference. Value can be obvious and objective, such as diamonds having more value than silver, and this could be for several reasons: scarcity of a resource, monetary cost, and perhaps demand. Value can be less obvious and subjective, such as when a person refuses to sell or give away an old, chipped tea set, or a baby blanket. What we e*stablish* as valuable could inform reasons for action. We have less reason to perform actions which are going to diminish that which we value and more reason to perform actions which will validate or confirm that which we value. To determine how to act in a way that will ensure we honor that which we find valuable, we could appeal to rationality norms to guide us.

For Frankfurt (1988), what is important for us is where we find reason for actions. It is what I directly care about that guides my actions. Frankfurt argues that “it is by caring about things that we infuse the world with importance” (Frankfurt, 2004, p. 23). When I confer value onto something, then it becomes normatively binding over my actions. Frankfurt (1988, p. 260) holds that a person identifies themselves with that which they care about in the sense that they make themselves vulnerable to losses and susceptible to benefits. If caring is that which creates value, then it is important that there is a set of norms– rationality norms– which can ensure that this *value-conferral* is always logically and rationally guided.

Gaut offers an alternative view - a *recognitional model* of practical reason (Gaut, 1997, p. 179). Gaut claims that we should be looking at recognitional models of value. The account Gaut offers sees action as responsive to value rather than value-conferring. Gaut further argues that value-responsiveness is not based primarily on rational choice, but also on this conception of a desire for human flourishing.

This paper sees it as a possibility that reasons can stem from both objective and subjective value^3^. We should not subsume both types of reasons under one account of value. In decision-making processes, there exists independent and objective reasons that apply to most. These types of reasons are better accounted for under the recognitional model of value. However, subjective reasons are arguably better accounted for under the conferral model.

## Voluntarist Reasons

How value is conferred or recognized tells an important part of the story of how we are able to will reasons. Ruth Chang^4^ (2009) asks us to imagine a world in which we are faced with two possible career choices– and you find yourself in the unique position to have equal reason to do both. First, you could choose to become a trapeze artist where you wear sequined leotards and perform daring exercises under a big top. Or second, you could become a philosopher where you spend your days in a less exciting pursuit of doing research (Chang, 2009, pp. 249–250).

She asks us to further imagine having weighed up every considerable reason for choosing either– essentially we are faced with a tie. Perhaps, “you have sufficient reason to choose among several alternatives or [your] choice is beyond the reach of practical reason” (Chang, 2009, p. 249). Given that this decision is an important one, far more important than the meal you would choose on a flight for example, she asks: “What should you do? It seems it would be a mistake for you to simply pick or plump for one career” over the other (Chang, 2009, p. 250).

Champagne (2015) argues that this type of situation (Buridan’s Ass) is an open challenge to rational decision-making: “given that an agent could conceivably confront equally attractive alternatives, it is an open question whether rational choice theory can ever eliminate indeterminacy” (Champagne, 2015, p. 127). After examining several theories of how one would deal with a Buridan’s Ass case^5^, Champagne concludes that either it is the case that utilities assigned to both options can never be truly equal, or that agents must appeal to a feature of non-reasoning faculty– such as the *will*– to deal with this. He claims that appealing to the will “is by no means a silly position” (2015, p. 146).

Stone (2014, p. 195) also argues that “invariably occasions arise in which the reasons known to the agent fail to single out a determinate option. When reasons cannot determine the option to select on their own, the agent must resort to some form of non-reasoned decision-making (NRDM) which include– picking, randomizing, deferring, and judging”– thereby reaching a similar conclusion to Champagne, that perhaps the only way out is through appealing to a feature of non-reasoned decision making.

Chang (2009) argues that we have three possible options. First, we could randomly pick one over the other - perhaps by way of flipping a coin. Chang argues, however, that with important decisions such as choosing between careers, randomly choosing could be detrimental and is not something we should do. Second, we could go back and examine our reasons one-by-one and hopefully something will tip the scales in favor of one over the other. Third, we could *will* an alternative (Chang, 2009, p. 253). This willing a reason is something Stone (2014) may argue is not within the realm of reasoned decision-making, and Champagne (2015) would likely agree.

Chang (2014) argues that this process *is rational* and that “when we choose between options that are on a par […] we can put ourselves behind an option… This response in hard choices is a rational response, but it’s not dictated by reasons given to us. Rather, it’s supported by reasons created by us”. The reasons created by us, importantly, allows us to exercise our normative powers, such that they hold normative force over our actions. This is argued through the distinction between voluntarist and non-voluntarist reasons.

Non-voluntarist reasons (or given reasons) are those which “we ordinarily take ourselves to have– reasons whose normativity derives either from normative reality or from our desires, but not from our own act of will” (Chang, 2009, p. 256). Conversely, “our voluntarist reasons […] are the reasons we create for ourselves by taking a consideration to be the reason when our given reasons have run out” (Chang, 2009, p. 256). If one is torn between being a philosopher or trapeze artist, and after they have carefully examined their reasons and are practically certain that they have run out of reasons, then they should take their secret love for sequins as a reason to choose trapezing over philosophy. “Through an act of will, [you can make it] a reason that [is] relevant to your choice” (Chang, 2009, p. 257).

Creating a voluntarist reason requires a certain buy-in from the individual that this consideration– not any other– is “a consideration that counts in favor of” some action or attitude (Scanlon, 2004)– in particular, this reason one may have willed as their reason counts in favor of a particular action. As discussed in the previous section, an individual is both capable of responding to, and conferring value. By taking a consideration as a voluntarist reason, we add normative weight to our reasons. We now have more overall reason to do the thing we have a voluntarist reason to do– whereas before, we had run out of our given reasons. “This willing creates normativity by creating new reasons whose normativity derives from the very act of will” (Chang, 2009, p. 255). There is an important distinction to note between merely plumping for a reason and willing a consideration to be a reason: through this act of willing, we are committing to an alternative as something we care about– following the line of argument of Frankfurt, it is what we care about where we can find normativity, such that “willing a consideration to be a reason is part of the process of making oneself into a distinctive normative agent, that is, creating one’s own ‘rational identity’ (Chang, 2009, p. 259).

*Willing* in decision making is still part of the story of rationality in that it provides me a reason that informs my decision making and actions. Raz (1999, p. 48) notes that

The will is the ability to choose and perform intentional actions. […] Commonly when we so choose, we do what we want, and we choose what we want, from among the most eligible options […], similarly, when faced with unpalatable but unavoidable and incommensurate options, […] it would be correct to say that I want to give up the one I choose to give up.

Three things to take away from the above:

First, that decision-making is partially constitutive of agency; we believe this is not controversial. Second (and perhaps a little controversial), is that voluntarist reasons (the version proposed by Chang) is partially constitutive of agential decision-making. Third, that most human agents are able to extract themselves out of ties– there’s a reason why “paralysis of action is not a pervasive phenomenon” (Champagne, 2015, p. 146) and this paper argues that voluntarist reasons might just be it.

## AI and Tie-Breaking

One promising (and perhaps too simple) way to measure the rationality capacity of an individual or entity is to determine the capacity of means-ends reasoning. Considering Chang’s account of voluntarist reasons, it appears though that means-ends reasoning is not always as simple as determining the best possible means to achieve the goals, especially when reasons to perform one action over another have run out, or when we are faced with a tie in the decision-making process. What further complicates the process of determining rational agency of an individual or entity is that the capacity to generate voluntarist reasons may be one of several hallmark features of exercising genuine agency. In this section, this paper examines the process of decision-making of artificial intelligence systems in situations of ties. It is believed that this process will inform the degree to which we can currently determine agency-capacity in artificial intelligence.

### Let’s Begin with a Scenario

Typically, current AI systems tasked with making decisions employ artificial neural networks, which are developed on a set of training data and then put to use evaluating actual scenarios (Reinhardt & Müller, 1990; Abiodun et al., 2018). Due to the design of these neural networks, ties frequently occur when evaluating possible choices. Strategies for breaking these ties are thus a significant design consideration when building AI systems and are a core part of how modern neural networks operate (Kuncheva, 2004).

Classifier neural networks, such as those driving decision making in the AI systems in wide use currently, are often an ensemble network (Kokkinos & Margaritis, 2014). In an ensemble network, multiple networks are each individually trained for the same purpose, but trained differently to the others. When making a decision, each individual network reaches its own conclusion on the task and ‘votes’ for what the ensemble’s decision should be. The decision rendered by the ensemble network is whichever conclusion was voted on by a plurality (often referred to as a “majority” in the literature) of the constituent networks (Kuncheva, 2004, pp. 12–125).

Consider how AI is currently being used in healthcare, particularly as a useful tool in medical diagnostics. An ensemble neural network can be trained to determine if a given tumor in an x-ray scan is benign or cancerous (Liu et al., 2018). In a binary classification such as this, avoiding ties is as simple as having an odd number of networks in the ensemble (Smith, 2022).

In situations that deal with non-binary classifications, breaking ties becomes increasingly difficult. Challenges arise when identifying objects in a photograph such that if a scenario requires an AI to determine if a particular object is a dog, a cat, a raccoon, or something else - this becomes more challenging. Challenges also abound when using algorithms in HR hiring processes. Which of the many applicants is best suited for the position? In this type of multi-class problem, the classifications reached by the individual networks will converge on a small number of high probability outcomes. Even a larger ensemble network containing ten or more individual neural networks cannot escape the problem of ties created by plurality voting systems (Smith, 2022).

Just as with humans, when a tie occurs, it must be broken. In AI, when a tie occurs, a classifier network finds a way to break the tie. Problematically, and which will be discussed in the next section, a classifier network is *simple* in the sense that it can perform only the specific classification task that it has been trained to do. It does not know *how* or *why* it is being used, nor can it evaluate the relative importance or immediacy of the decision it has been tasked to make. Some early neural networks were designed to return errors (Dudani, 1976) (i.e. make no determination) in the case of ties, but this was soon criticized (Bailey & Jain, 1978) and including tie-breaking steps is a core part of neural network design (Kuncheva, 2004, p. 195) (Kokkinos & Margaritis, 2014).

### How the Ties are Broken in AI Decision-Making

There are several strategies used for breaking ties within neural networks, and current neural networks utilize one or more of these strategies. Viewed broadly, the various strategies for breaking ties can be understood as relying on (i) logic, (ii) math, (iii) arbitrary factors or (iv) randomness. Breaking ties using arbitrary or random strategies is often used as a last resort if a tie still exists after using a more robust tie-breaking method based on logic or math (Tahir, 2012).

Consider the example of a classifier ensemble neural network that identifies the species of animals in photos, as introduced above, and assume that the result of plurality voting within the ensemble network has produced a tie where an equal number of votes exist for both ‘dog’ and ‘cat’. The tie can be broken by making logical assumptions based on the relative frequency of dogs and cats in the training data. If dogs appeared more frequently than cats in the training data, then the network could conclude that the current image is more likely to contain a dog by assuming that the relative distribution of dogs and cats in the current data will be similar to the distribution found in the training data. Alternatively, by similar logic, the network could break the tie by considering the relative frequency of dogs and cats present in already analyzed images from the set of data currently being analyzed (Fürnkranz, 2002).

Another type of tie-breaking strategies instead perform mathematical analysis on the tied categories. One such technique is to make use of aggregated confidence from the individual networks (Schapire & Singer, 1999). Within individual neural networks, each node in the network chooses to activate or not, based on the inputs from the nodes it is connected to. This activation then possibly triggers activation of other connected nodes, not unlike how a neuron fires or does not based on the activity of other neurons that it is connected to. With each activation or non-activation, the node does so based on its degree of confidence as to whether it should activate. (“Do I see dog-like features in this image or not?”) A decision to activate or not is binary, but confidence regarding this decision can be any value from 0.000 to 1.000, with exact precision varying for each network. These confidence values can be averaged across each node in the network to produce a confidence value along with the decision (Schapire & Singer, 1999).

A nearest neighbour comparison is another commonly used mathematical approach to breaking ties. To continue with the animal identification example from above: One technique for training a neural network to analyze these images would compare a target image that may contain an object, for example a dog, to the existing set of images the network was trained on in order to find images that contain similar objects. If a plurality of the most similar images is known to contain a dog, then the network determines that the object in the target image also belongs to that category. This technique is known as the *k*-nearest neighbour algorithm (“k-NN”), where *k* is a number chosen by the network designers (Wu et al., 2007). To break a tie, the same core principle can be followed, but on a smaller scale. In a single nearest neighbour classification (“1-NN”), the network determines which single image from the known images is most similar to the target image. If a tie remains, a 2-NN classification can be done, then 3-NN, etc. (Kuncheva, 2004, pp. 56–67).

If the above tie-breaking strategies fail, arbitrary tie-breaking may be employed (cf. Tahir, 2012; Kokkinos & Margaritis, 2014). Arbitrary tie-breaking uses some property of the networks or classes themselves. For instance, the individual networks within an ensemble network have an index associated with them, and this index is used to refer to each network within the programming code. (Network #1, network #2, network #3, etc.) The classes that objects can belong to similarly have their own index, with each class having its own number. One way to break ties is to choose the network (from among the ones involved in the tie) which has the lowest or highest index value.

Random tie breaking simply involves generating random numbers for the voting networks involved in the tie and chooses the one with the lowest or highest random value (cf. Fürnkranz, 2002; Kokkinos & Margaritis, 2014). Random tie breaking is distinct from arbitrary tie breaking. For any given tie scenario, an arbitrary algorithm will produce a knowable, predictable result each time that scenario occurs. A random algorithm will not produce a predictable result.

To summarise, tie-breaking methods for a neural network can be based on logic, such as knowledge of the known frequency of classes in the training dataset or the target dataset. It can also be based on math, such as using combined confidence coefficients (e.g. activation weights) for each node or using a k-NN algorithm. When these methods fail to break a tie, a decision can be reached arbitrarily, such that outputs from identical tie-break scenarios will be consistent, or randomly, such that outputs from identical tie-break scenarios will be unpredictable.

## The Impacts of AI Tie-Breaking on the Agency-Status of Machines

In Sect. 4, we examined strategies that AI may employ in the event of ties in the decision-making process. We focused primarily on the scenario of identification of animals in photographs. Note, however, that the decision-making process potentially looks the same even if we increase the stakes of the outcome of the decision-making process. For example, the same types of decision-making strategies may apply to HR algorithmic hiring (Kearn & Roth, 2020) or perhaps even the implementation of autonomous vehicles or automation processes in manufacturing (Acemoglu & Autor, 2011; Blit, 2020; Frey & Osborne, 2017; Guenat et al., 2022; Santos et al., 2015). It goes without saying that the stakes in hiring the right candidate or the safe operation of autonomous vehicles looks quite different from the stakes in identifying images in a picture (unless of course, the identification could lead to harm - such as bias) (Kearns & Roth, 2020).

If we begin to recognise that the same strategies are potentially employed across different levels of decision-making, then we become more aware of multiple concerns that may arise from this, such as “the problem […] that the training data used in machine learning applications can often contain all kinds of hidden (and-not-so-hidden) biases, and the act of building complex models from such data can both amplify these biases and introduce new ones” (Kearns & Roth, 2020, p. 61). In this section, we perform a comparative analysis between human tie-breaking processes and AI tie-breaking processes. We proceed to analyse what the differences mean in terms of the agency-status of machines.

In sections “Agency in AI, and in Humans” and “Voluntarist Reasons” we unpacked what decision-making looks like for humans in the event of a tie. We showed that means-ends reasoning (at a minimum) underpins agency and that decision making is an important part of this process. We also indicated that value is either responsive or conferred and this plays a pivotal role in the ability for human agents to will a reason in favour of one choice over another in the case of a tie. In the above section, we discuss the most common strategies AI can use in the face of a tie: (i) logic, (ii) mathematics, (iii) arbitrariness, (iv) randomness. For ease of discussion, let’s label these as AI(i), AI(ii), AI(iii), AI(iv) respectively.

For Chang (2009), all of these would be perfectly acceptable if we were choosing between chicken or fish as our meal of choice on a flight. The choice is low-stakes enough that plumping for one over the other will result in very little harm, or even benefit. In the event of a large-stakes choice however, it is likely that Chang would take issue with a few of these strategies. Let’s use the example of HR algorithmic hiring. The stakes of hiring the right person for the job is twofold: will the person be the right fit in the company, and will the candidate be treated fairly and receive proper consideration? Let’s imagine that two candidates from similar education, demographic, socio-economic, and religious backgrounds apply for the same position. They are both, essentially, a perfect fit. The AI is faced with a tie.

This decision is high-stakes enough that Chang would argue that plumping for a reason would not be advisable, nor the best course of action. Humans would go over all the details again until they are sure that there is no reason in favour of one over the other (Chang, 2009). The human HR manager may decide that the one candidate’s membership to a debate club is a reason for them to choose candidate A over candidate B. This voluntarist reason then provides a normative force that pushes her to choose candidate (A) Is candidate A the best choice? Perhaps not. But it’s enough to make the decision. One may argue that being a debater is an arbitrary consideration to will it as a reason to choose candidate A over candidate (B) Indeed, it may seem arbitrary to an observer, but for the HR manager, this becomes *her reason* (value conferral) to choose candidate A, and it becomes the reinforcing reason for her to act in favour of choosing A and supporting this decision (at least to herself). This is her practicing her agency to the fullest extent.

Under the same scenario, we will immediately exclude AI(iii), AI(iv) from consideration. We exclude AI(iii) and AI(iv) because this is a high-stakes choice and plumping for one of the other is not what we want. Plurality voting within ensemble classifier neural networks (specifically AI(i) and AI(ii) may offer a reasonable analogue for human agent decision-making and produces scenarios where humans may be in the position to *will* a voluntarist reason to break a tie. As a human agent would view or weigh an important decision from multiple perspectives, each constituent network within an ensemble network independently analyzes an input. As a human agent would then combine the results of each perspective, an ensemble network combines the results by having each constituent network vote for a potential output. As a human agent may conclude that two or more options are equally good, votes in an ensemble network may produce a tie between two or more potential outputs. As a human agent would seek a way to resolve the impasse and make a decision, a neural network uses one or more methods to break a tie. But, arguably, unlike a human agent, AI cannot *will* a non-objective reason to serve as a reason to break a tie.

For Velleman, agents pursue that which is most intelligible to do. Intelligibility for an agent, requires, at least in some sense, making sense of oneself in the world. Velleman suggest we (2013, p. 32), “keep in mind that self-understanding is not simply a matter of making sense of oneself as one is; it is also a matter of making sense to oneself”. In the creation of an agent we would include not only the ability for practical reasoning (in the form of rationality), but also we would need to include a drive or desire to make sense of the world. The drive or desire to make sense would be that which ensures intelligibility. Intelligibility is that which allows us to understand ties and having equal reasons to choose one option or another, it also affords us the ability to choose a subjective reason as one to break ties.

For Velleman, “a rational agent tends to enact the attitudes and traits that he conceives himself to have, by pursuing what he thinks that he wants, through means in which he thinks that he believes, and in ways characteristic of other dispositions that he ascribes to himself” (Velleman, 2013, p. 90). Three important aspects then: first, that an agent has attitudes and traits necessary for self-conception; second, that an agent is able to, and is free to pursue what he wants; third, that the agent understands and identifies certain descriptions or concepts that describe their identity. This would assume, at a minimum, a sense of self or ‘I’.

The first may be answered by studies done in ‘Sense of Agency’ (SoA) in machines. Considerable advancements have been made in this field. SoA has been proposed as fundamental to the experience of self-awareness, volition, and understanding causal structure in the world. According to Legaspi et al. (2019, p. 84), “concrete implementations [in AI] are limited so far. Most can be seen in cognitive developmental robotics, where the robot distinguishes itself from the world to enhance its motor and cognitive skills through sensorimotor predictive processes”. Importantly, it was also noted that “a full account of SoA should also consider non-sensorimotor cues (e.g. background beliefs and environmental cues) and ad hoc reasoning” (Legaspi et al., 2019, p. 84)– which is currently lacking in AI (and may very well be imbedded in the second and third aspect).

For the second and third aspect:

The question of how and by what means swimming is performed is not crucial to the concept of swimming. But the difference between a being that thinks I think, or at least between one that feels I feel, or that experiences I want, and a machine that has no such self-consciousness, is so enormous that those terms (to think, to feel, to want) should not be used for beings that have no I. To say that a computer feels is like saying that a planet flies just because it moves through space. (Schönecker, 2022, p.186)

## Conclusion

As technology develops at an unprecedented rate, especially artificial intelligence, such as Generative AI, it is our ethical and moral responsibility (Swanepoel, 2021b) to accurately determine the agency-status of machines. Agents are afforded certain rights and considerations, but also, and more importantly, there are expectations of the correct and acceptable ways for an agent to act. By setting up criteria to determine agency-status, we are one step closer to understanding how it is we should treat and behave towards machines and what kind of actions we should expect, and deem as acceptable by machines.

In this paper, we set up a hallmark feature of agency as the ability to break ties by generating voluntarist reasons. By examining Ruth Chang’s (2009) theory of voluntarist reasons, this paper concludes that agents, when faced with a tie, have the ability to will a reason to be that which allows them, or drives them, to break the tie and choose one option over another. In tie-breaking situations, AI predominantly uses strategies such as (i) logic, (ii) mathematics, (iii) arbitrariness, (iv) randomness. In this paper, we determine that these strategies are not adequate to will a reason in the event of a tie.

As AI researchers move forward in developing machines which act in the world and continue working towards imbuing AI with agency (or the sense thereof), we pose a challenge to AI researchers: to fully imbue AI with agency, you may need a full description of what agency entails and have a full repertoire of features of agency. Here we offer just one hallmark feature of agency to add to the list: that of the ability to break ties through generating voluntarist reasons.

## Footnotes

1 For more on the problem of anthropocentric definitions or ‘handling’ of evaluating agency, see Gunkel (2017); Floridi and Sanders (2004); Johnson and Miller (2008); Sullins (2006).

2 We have considered that determinists would argue against the notion of autonomous, intentional action as is described here– however, given the goal of this paper, we believe diving into discussions about free-will, determinism, and compatibilism is beyond the scope of this paper. See Dennett (1984); Frankfurt (1971); Huffer (2007); Lewis (1979); Wallace (1999), and for more on determinism, free-will, and compatibilism.

3 Note, if the philosophical views of how value informs our decision-making is not convincing to the reader, we can also appeal to neurobiology to show how value informs decision making, for more on this, see Damasio (2012).

4 It may be pertinent to point out that Sartre (1957) may have been a viable alternative to Chang for this paper. While we agree that Sartre does more to address the concerns of free will in decision making and does more to show that we perform actions which we take to be valuable and worthwhile, the authors believe that Chang’s Hybrid Voluntarism does more to show ownership and agency in decision-making in the event of a tie. We thank the anonymous reviewer for suggesting Sartre as an alternative to Chang.

5 Thanks to the anonymous reviewer who pointed us in this direction.
